# Genome sequences of *Bacteria* and *Archaea* published outside of *Standards in Genomic Sciences,* June – September 2011

**DOI:** 10.1601/sigs.2324675

**Published:** 2011-09-23

**Authors:** Oranmiyan W. Nelson, George M. Garrity

**Affiliations:** 1 Editorial Office, Standards in Genomic Sciences and Department of Microbiology, Michigan State University, East Lansing, MI, USA

## Abstract

The purpose of this table is to provide the community with a citable record of publications of ongoing genome sequencing projects that have led to a publication in the scientific literature. While our goal is to make the list complete, there is no guarantee that we may have omitted one or more publications appearing in this time frame. Readers and authors who wish to have publications added to this subsequent versions of this list are invited to provide the bibliometric data for such references to the SIGS editorial office.

**Phylum *Crenarchaeota***
**Phylum *Euryarchaeota***
*Pyrococcus yayanosii* CH1, sequence accession CP002779 [1]
*Methanocella paludicola*, sequence accession AP011532 [2]
*Halorhabdus tiamatea*, sequence accession AFNT00000000 [3]
*Thermococcus* sp. Strain 4557, sequence accession CP002920 [4]
**Phylum *Chloroflexi***
**Phylum *Proteobacteria***
*Ralstonia solanacearum* strain Po82, sequence accession CP002819 (chromosome) and CP002820 (megaplasmid) [5
*Desulfovibrio alaskensis* G20, sequence accession CP000112 [6]
*Methylophaga aminisulfidivorans* MP^T^, sequence accession AFIG00000000 [7]
*Acinetobacter* sp. P8-3-8, sequence accession AFIE00000000 [8]
*Sphingomonas* strain KC8, sequence accession AFMP01000000 [9]
*Brucella pinnipedialis* B2/94, sequence accession CP002078 and CP002079 [10]
*Salmonella enterica* Serovar Typhimurium UK-1, sequence accession CP002614 (chromosome), CP002615 (plasmid) [11]
*Bordetella pertussis* CS, sequence accession CP002695 [12]
*Alteromonas* sp. Strain SN2, sequence accession CP002339 [13]
*Escherichia coli* O104:H4, sequence accession AFOB00000000 (LB226692) and AFPS00000000 (01-09591) [14]
*Acidithiobacillus caldus*, sequence accession CP002573 (Chromosome), CP002574 (pLAtcm), CP002575 (pLAtc1), CP002576 (pLAtc2), CP002577 (pLAtc3) [15]
*Cupriavidus necator* N-1, sequence accession CP002877 (chromosome 1), CP002878 (chromosome 2), CP002879 (pBB1), and CP002880 (pBB2) [16]
*Oligotropha carboxidovorans* OM4, sequence accession CP002821 (OM4 chromosome), CP002822 (pHCG3b), CP002823 (pOC167B) [17]
*Oligotropha carboxidovorans* OM5, sequence accession CP002826 (OM5 chromosome), CP002827 (pHCG3), and CP002828 (pOC167) [17]
*Pantoea ananatis* LMG20103, sequence accession CP001875 [18]
*Helicobacter bizzozeronii* strain CIII-1, sequence accession FR871757 (chromosome) and FR871758 (HBZ-1) [19]
*Vibrio anguillarum* 775, sequence accession CP002284 to CP002285 [20]
*Zymomonas mobilis* subsp. pomaceae, sequence accession CP002865 (chromosome), CP002866 (p29192_1), CP002867 (p29192_2) [21]
*Agrobacterium* sp. strain ATCC 31749, sequence accession AECL01000000 [22]
*Xanthomonas* spp. strain Xrc, sequence accesssion CP002789 [23]
*Xanthomonas* spp. strain Xoc, sequence accesssion AAQN00000000 [23]
*Glaciecola* sp. Strain 4H-3-7+YE-5, sequence accession CP002526 (chromosome) and CP002527 (plasmid) [24]
*Escherichia coli* Strain HM605, sequence accession CADZ01000001 through CADZ01000154 [25]
*Salinisphaera shabanensis*, sequence accession AFNV00000000 [26]
*Methyloversatilis universalis* FAM5^T^, sequence accession AFHG00000000 [27]
*Alicycliphilus denitrificans* Strain BC, sequence accession CP002449 (chromosome), CP002450 (megaplasmid), CP002451 (plasmid) [28].
*Alicycliphilus denitrificans* K601^T^, sequence accession CP002657 (chromosome) and CP002658 (plasmid) [28]
*Oligotropha carboxidovorans* Strain OM4, sequence accession CP002821 (chromosome), CP002822 (pHCG3b), CP002823 (pOC167B) [29]
Oligotropha carboxidovorans Strain OM5, sequence accession CP002826 (chromosome), CP002827 (pHCG3), and CP002828 (pOC167) [29]
*Bradyrhizobiaceae* strain SG-6C, sequence accession AFOF01000000 [30]
*Hyphomicrobium* sp. Strain MC1, sequence accession FQ859181 [31]
*Shewanella* sp. Strain HN-41, sequence accession AFOZ01000000 [32]
*Myxococcus fulvus* HW-1, sequence accession CP002830 [33]
*Nitrosomonas* sp. Strain AL212, sequence accession NC_015222 (chromosome), NC_015223 pNAL21201), NC_015221 (pNAL21202) [34]
*Ruegeria* sp. Strain KLH11, sequence accession ACCW00000000 [35]
*Acidovorax avenae* subsp. avenae RS-1, sequence accession AFPT01000000 [36]
*Escherichia coli* (ExPEC), sequence accession AFAT00000000 [37]
*Vibrio mimicus* SX-4, sequence accession ADOO01000000 [38]
*Agrobacterium tumefaciens* Strain F2, sequence accession AFSD00000000 [39]
*Pasteurella multocida* subsp. gallicida AFRR01000001 to AFRR01000489 [40]
*Pseudomonas aeruginosa* 138244, sequence accession AEVV00000000 [41]
*Pseudomonas aeruginosa* 152504, sequence accession AEVW00000000 [41]
*Campylobacter jejuni* strain 305, sequence accession ADHL00000000 [42]
*Campylobacter jejuni* strain DFVF1099, sequence accession ADHK00000000 [42]
*Xanthomonas campestris pv. raphani strain 756C*, sequence accession CP002789 [43]
*Xanthomonas campestris pv. raphani strain BLS256*, sequence accession AAQN01000001 [43]
*Rickettsia heilongjiangensis*, sequence accession CP002912 [44]
*Acidiphilium* sp. Strain PM (DSM 24941), sequence accession AFPR00000000 [45]
*Pseudomonas putida* Strain S16, sequence accession CP002870 [46]
*Acinetobacter lwoffii*, sequence accession AFQY01000000 [47]
**Phylum *Firmicutes***
*Caldalkalibacillus thermarum* strain TA2.A1, sequence accession AFCE00000000 [48]
*Listeria monocytogenes* Scott A, sequence accession AFGI00000000 [49]
*Lactococcus garvieae* 8831, sequence accession AFCD00000000 [50]
*Natranaerobius thermophilus* JW/NM-WN-LF, sequence accession CP001034 (chromosome), CP001035 (plasmid) [51]
*Melissococcus plutonius* ATCC 35311, sequence accession AP012200 (chromosome) and AP012201 (plasmid) [52]
*Lactobacillus buchneri* NRRL B-30929, sequence accession CP002652 (chromosome), CP002653 (plasmid pLBU01), CP002654 (plasmid pLBU02), and CP002655 (plasmid pLBU03) [53]
*Lactobacillus kefiranofaciens* ZW3 , sequence accession CP002764 (chromosome), CP002765 (plasmid), and CP002766 (plasmid) [54]
*Bacillus megaterium* strain QM B1551, sequence accession CP001983 (chromosome), CP001984 to CP001990 (plasmids pBM100 through pBM700) [55]
*Bacillus megaterium* strain DSM319, sequence accession CP001982 (chromosome) [55]
*Listeria monocytogenes* serovar 4a strain M7, sequence accession CP002816 [56]
*Bacillus coagulans* 2-6, sequence accession CP002472 [57]
*Streptococcus salivarius* strain CCHSS3, sequence accession FR873481 [58]
*Paenibacillus elgii* B69, sequence accession AFHW01000000 [59]
*Lactobacillus pentosus* MP-10, sequence accession FR871759 through FR871848 [60]
*Leuconostoc pseudomesenteroides* KCTC 3652, sequence accession AEOQ00000001 through AEOQ00001160 [61]
*Lactobacillus mali* KCTC 3596, sequence accession BACP01000001 through BACP01000122 [62]
*Paenibacillus polymyxa* Type Strain ATCC 842^T^, sequence accession AFOX01000000 [63]
*Streptococcus salivarius* strain JIM8777, sequence accssion FR873482 [64]
*Lactobacillus cypricasei* KCTC 13900, sequence accession BACS01000001 to BACS01000487 [65]
*Lactobacillus zeae* KCTC 3804, sequence accession BACQ01000001 to BACQ101000113 [66]
*Listeria monocytogenes* Serovar 4a Strain M7, sequence accession CP002816 [67]
*Lactobacillus salivarius* GJ-24, sequence accession AFOI00000000 [68]
*Lactobacillus johnsonii* PF01, sequence accession AFQJ01000000 [69]
*Clostridium acetobutylicum* DSM 1731, sequence accession CP002660 through CP002662 [70]
*Lactobacillus suebicus* KCTC 3549, sequence accession BACO01000000 [71]
*Brevibacillus laterosporus* LMG 15441, sequence accession AFRV00000000 [72]
*Lactobacillus salivarius* NIAS840, sequence accession AFMN00000000 [73]
*Bifidobacterium animalis* subsp. lactis CNCM I-2494, sequence accession CP002915 [74]
*Megasphaera elsdenii*, sequence accession HE576794 [75]
*Lactobacillus versmoldensis* KCTC 3814, sequence accession BACR01000001 to BACR01000102 [76]
*Lactobacillus pentosus* IG1, sequence accession FR874848 to FR874860 [77]
*Alicyclobacillus acidocaldarius* Strain Tc-4-1, sequence accession CP002902 [78]
*Streptococcus thermophilus* Strain JIM8232, sequence accession FR875178 [79]
*Streptococcus equi subsp. zooepidemicus* Strain ATCC 35246, sequence accession CP002904 [80]
*Bacillus amyloliquefaciens* XH7, sequence accession CP002927 [81]
*Leuconostoc kimchii* Strain C2, sequence accession CP002898 [82]
*Lactobacillus malefermentans* KCTC 3548, sequence accession BACN01000001 to BACN01000172 [83]
*Weissella koreensis* KACC 15510, sequence accession CP002900 [84]
**Phylum *Tenericutes***
*Mycoplasma bovis* Strain Hubei-1, sequence accession CP002513 [85]
*Mycoplasma fermentans* Strain M64, sequence accession NC_014921 [86]
*Haloplasma contractile*, sequence accession AFNU00000000 [87]
*Mycoplasma ovipneumoniae* Strain SC01, sequence accession AFHO01000000 [88]
**Phylum *Actinobacteria***
*Kocuria rhizophila* P7-4, sequence accession AFID00000000 [89]
*Streptomyces* S4, sequence accession CADY01000000 [90]
*Corynebacterium nuruki* S6-4^T^, sequence accession AFIZ00000000 [91]
*Propionibacterium humerusii*, sequence accession AFAM00000000.1 [92]
Strain JDM601, sequence accession CP002329 [93]
*Streptomyces* sp. strain Tü6071, sequence accession AFHJ01000000 [94]
*Bifidobacterium breve* UCC2003, sequence accession CP000303 [95]
*Propionibacterium acnes*, sequence accession CP002815 [96]
*Amycolicicoccus subflavus* DQS3-9A1^T^, sequence accession CP002786 (chromosome), CP002787 (plasmid pAS9A-1), and CP002788 (plasmid pAS9A-2). [97]
*Gordonia neofelifaecis* NRRL B-59395, sequence accession AEUD01000000 [98]
*Pseudonocardia dioxanivorans* strain CB1190, sequence accession NC_015312-4 and CP002595-7 [99]
*Bifidobacterium longum* subsp. *longum* KACC 91563, sequence accession  CP002794 to CP002796 [100]
*Streptomyces cattleya* NRRL 8057, sequence accession FQ859185 (chromosome) and FQ859184 (megaplasmid) [101]
*Rhodococcus* sp. Strain R04, sequence accession AFAQ01000000 [102]
*Mycobacterium bovis* BCG Moreau, sequence accession [103]
*Saccharopolyspora spinosa* NRRL 18395, sequence accession [104]
*Mycobacterium tuberculosis* CCDC5079, sequence accession [105]
*Mycobacterium tuberculosis* CCDC5180, sequence accession [105]
*Amycolatopsis mediterranei* S699, sequence accession CP002896 [106]
*Nesterenkonia* sp. Strain F, sequence accession AFRW01000000 [107]
*Streptomyces xinghaiensis* NRRL B24674^T^, sequence accession AFRP01000000 [108]
**Phylum *Chlamydiae***
*Chlamydophila abortus* variant strain LLG, sequence accession AFHM01000000 [109]
*Chlamydia psittaci* 6BC, sequence accession CP002586 (chromosome), CP002587 (plasmid) [110]
*Chlamydia psittaci* Cal10, sequence accession AEZD00000000 (draft chromosome and plasmid) [110]
*Chlamydia trachomatis*, sequence accession CP002024 [111]
**Phylum *Spirochaetes***
*Spirochaeta thermophila* DSM 6192, sequence accession CP001698 [112]
*Brachyspira intermedia*, sequence accession CP002874 (chromosome) and CP002875 (plasmid) [113]
**Phylum *Fibrobacteres***
**Phylum *Bacteroidetes***
*Porphyromonas gingivalis* TDC60, sequence accession AP012203 [114]
*Krokinobacter* sp. strain 4H-3-7-5, sequence accession CP002528 [115]
*Lacinutrix* sp. strain 5H-3-7-4, sequence accession CP002825 [115]
*Bacterium* HQM9, sequence accession AFPB00000000 [116]
*Anaerophaga* sp. Strain HS1, sequence accession AFSL00000000 [117]
*Capnocytophaga canimorsus* Strain 5, sequence accession CP002113 [118]
*Mesoflavibacter zeaxanthinifaciens* strain S86, sequence accession AFOE00000000 [119]
**Phylum *Verrucomicrobia***
**Phylum *Lentisphaerae***
**Phylum *Thermotogae***
*Kosmotoga olearia* Strain TBF 19.5.1, sequence accession CP001634 [120]
**Domain *Archaea***
"Candidatus *Nitrosoarchaeum koreensis*" MY1, sequence accession AFPU00000000 [121]

**Non-Bacterial genomes**
North-European Cucumber *Cucumis sativus* L., sequence accession FI132140–FI136208, GS765762–GS766880, GS815969–GS874855 [122]
Castor bean *Ricinus communis* organelle genome, sequence accession JF937588(chloroplast), HQ874649 (mitochondria) [123]
Stretch Lagoon Orbivirus *Umatilla*, sequence accession HQ842619 through HQ842628 [124]
Atlantic cod *Gadus morhua*, sequence accession CAEA01000001 through CAEA01554869 [125]
Potato *Solanum tuberosum* L., sequence accession  GS025503 through GS026177 [126]
ΦCA82, sequence accession HQ264138 [127]
*Paramecium caudatumreveals* mitochondria, sequence accession NC001324 [128]
bacteriophage IME08, sequence accession NC_014260 [129]
virus (ILTV), sequence accession HQ_630064 [130]
Australian kangaroo *Macropus eugenii*, sequence accession ABQO000000000 [131]
Aichi virus, sequence accession FJ890523 [132]
"Candidatus Tremblaya princeps" Strain PCVAL, sequence accession CP002918 [133]
